# Manic episode in a patient with pancreatic adenocarcinoma: a case report

**DOI:** 10.1192/j.eurpsy.2024.910

**Published:** 2024-08-27

**Authors:** J. I. Mena Garcia, H. Andreu, S. Salmeron, I. Ochandiano, E. Cesari

**Affiliations:** Psychiatry and Psychology Service, Hospital Clínic, Barcelona, Spain

## Abstract

**Introduction:**

Psychiatric comorbidity is common in cancer patients, emphasizing the need for comprehensive care. While depressive symptoms in pancreatic cancer (PC) have been studied, there is limited attention given to manic symptoms. This case report aims to contribute to the knowledge of PC psychiatric comorbidities by describing a case of a 61-year-old patient with stage IV PC, with no personal or family psychiatric history, who presented a sudden onset manic episode.

**Objectives:**

Our goal is to contribute to the growing knowledge of psychiatric comorbidities of PC focusing on manic symptoms by describing the case of a patient with stage IV PC without previous psychiatric history who presented a sudden onset of a manic episode.

**Methods:**

We describe the mentioned clinical case. We also searched for previous case reports of maniac episodes in pancreatic cancer using a PubMed query.

**Results:**

The patient, a 61-year-old male with stage IV PC, presented at the Emergency Room with abrupt behavioural changes suggestive of a manic episode of two weeks of evolution. The patient had been undergoing chemotherapy and short 3-day cycles of corticosteroids for the past 9 months but had been off this treatment for 20 days when the episode began. Acute organic causes were ruled out. The patient was admitted to the psychiatric unit, where organic screening was expanded and treatment with antipsychotics and a mood stabilizer was initiated with subsequent remission of symptoms after two weeks.

This article describes the case of a man with a PC diagnosis who had no prior psychiatric history and was admitted to the inpatient psychiatry unit due to a manic episode involving high-risk behavioral disturbances and megalomaniac psychotic symptoms. Several factors may have contributed to the onset of these symptoms, including corticosteroid use after chemotherapy and certain chemotherapy agents. However, due to temporal factors, these factors do not fully explain the episode.

The exact biological mechanisms behind the manic symptoms remain unknown, but hypotheses include gene-environment interactions in bipolar disorder and immunodysregulation related to the production of inflammatory cytokines. We found in the literature four cases that have reported new-onset mania as an initial symptom of PC, but the causal relationship is unclear.

**Conclusions:**

Notably, this case differs from others due to the rapid remission of symptoms and the use of lithium therapy. While the underlying mechanisms are still unclear, this case contributes to understanding this rare complication of PC and may help in developing consensus on clinical management. Future research will further explore the pathophysiology of psychiatric symptoms in PC and appropriate therapeutic approaches.

This case shows a manic episode as a rare psychiatric complication in PC. In the literature reviewed, four other similar cases have been observed.

**Disclosure of Interest:**

None Declared

